# On the effectiveness of compact biomedical transformers

**DOI:** 10.1093/bioinformatics/btad103

**Published:** 2023-02-24

**Authors:** Omid Rohanian, Mohammadmahdi Nouriborji, Samaneh Kouchaki, David A Clifton

**Affiliations:** Department of Engineering Science, University of Oxford, Oxford, UK; NLPie Research, Oxford, UK; NLPie Research, Oxford, UK; Department of Electrical and Electronic Engineering, University of Surrey, Guildford, UK; Department of Engineering Science, University of Oxford, Oxford, UK; Oxford-Suzhou Centre for Advanced Research, Suzhou, China

## Abstract

**Motivation:**

Language models pre-trained on biomedical corpora, such as BioBERT, have recently shown promising results on downstream biomedical tasks. Many existing pre-trained models, on the other hand, are resource-intensive and computationally heavy owing to factors such as embedding size, hidden dimension and number of layers. The natural language processing community has developed numerous strategies to compress these models utilizing techniques such as pruning, quantization and knowledge distillation, resulting in models that are considerably faster, smaller and subsequently easier to use in practice. By the same token, in this article, we introduce six lightweight models, namely, BioDistilBERT, BioTinyBERT, BioMobileBERT, DistilBioBERT, TinyBioBERT and CompactBioBERT which are obtained either by knowledge distillation from a biomedical teacher or continual learning on the Pubmed dataset. We evaluate all of our models on three biomedical tasks and compare them with BioBERT-v1.1 to create the best efficient lightweight models that perform on par with their larger counterparts.

**Results:**

We trained six different models in total, with the largest model having 65 million in parameters and the smallest having 15 million; a far lower range of parameters compared with BioBERT’s 110M. Based on our experiments on three different biomedical tasks, we found that models distilled from a biomedical teacher and models that have been additionally pre-trained on the PubMed dataset can retain up to 98.8% and 98.6% of the performance of the BioBERT-v1.1, respectively. Overall, our best model below 30 M parameters is BioMobileBERT, while our best models over 30 M parameters are DistilBioBERT and CompactBioBERT, which can keep up to 98.2% and 98.8% of the performance of the BioBERT-v1.1, respectively.

**Availability and implementation:**

Codes are available at: https://github.com/nlpie-research/Compact-Biomedical-Transformers. Trained models can be accessed at: https://huggingface.co/nlpie.

## 1 Introduction

There has been an ever-increasing abundance of medical texts in recent years, both in private and public domains, which provide researchers with the opportunity to automatically process and extract useful information to help develop better diagnostic and analytic tools (Locke *et al.*, 2021). Medical corpora can come in various forms, each with its own specific context. These include electronic health records, medical texts on social media, online knowledge bases and scientific literature (Kalyan and Sangeetha, 2020).

With the advent of the transformers architecture (Vaswani *et al.*, 2017), the natural language processing (NLP) community has moved towards utilizing pre-trained models that could be used as a strong baseline for different tasks and also serve as a backbone to other sophisticated models. The standard procedure is to use a general model pre-trained on a very large amount of unstructured text and then fine-tune the model and adapt it to the specific characteristics of each task. Most state-of-the-art NLP models are based on this procedure.

A related alternative to the standard pre-train and fine-tune approach is domain-adaptive pretraining, which has been shown to be effective on different textual domains. In this paradigm, instead of fine-tuning the pre-trained model on the task-specific labelled data, pre-training continues on the unlabelled training set. This allows a smaller pre-training corpus, but one that is assumed to be more relevant to the final task (Gururangan *et al.*, 2020). This method is also known as continual learning, which refers to the idea of incrementally training models on new streams of data while retaining prior knowledge (Parisi *et al.*, 2019).

NLP researchers working with biomedical data have naturally started to incorporate these techniques into their models. Apart from vanilla fine-tuning on medical texts, specialized BERT-based models have also been developed that are specifically trained on medical and clinical corpora. ClinicalBERT (Huang *et al.*, 2019), SciBERT (Beltagy *et al.*, 2019) and BioBERT (Lee *et al.*, 2020) are successful attempts at developing pre-trained models that would be relevant to biomedical NLP tasks. They are regularly used in the literature to develop the latest best performing models on a wide range of tasks.

Regardless of the successes of these architectures, their applicability is limited because of the large number of parameters they have and the amount of resources required to employ them in a real setting. For this reason, there is a separate line of research in the literature to create compressed versions of larger pre-trained models with minimal performance loss. DistilBERT (Sanh *et al.*, 2019), MobileBERT (Sun *et al.*, 2020) and TinyBERT (Jiao *et al.*, 2020) are prominent examples of such attempts, which aim to produce a lightweight version of BERT that closely mimics its performance while having significantly less trainable parameters. The process used in creating such models is called distillation (Hinton *et al.*, 2015).

Compact models allow faster training and inference which is highly desirable in low-power settings such as mobile devices or when processing large volumes of data that would take much longer with a full-sized model. Low-resource hospitals or clinics, especially in the developing world, can benefit from capable and lightweight models that could be used in diagnosis support or risk prediction, and the reduced computational and memory requirements of a compact model may be worth the trade-off in accuracy in such environments. For biomedical applications, there are cases where the performance of a compact language model may be sufficient for a given task, even if performance may not be as high as a larger model. For example, a compact model may be able to achieve acceptable accuracy for a binary classification task, even if it does not perform as well as a larger model on more complex tasks. Techniques such as distillation from larger language models which is explored in this work mitigate the performance trade-off associated with using a compact model.

In this work, we first train three distilled versions of the BioBERT-v1.1 using different distillation techniques, namely, DistilBioBERT, CompactBioBERT and TinyBioBERT. Following that, we pre-train three well-known compact models (DistilBERT, TinyBERT and MobileBERT) on the PubMed dataset using continual learning. The resultant models are called BioDistilBERT, BioTinyBERT and BioMobileBERT. Finally, we compare our models to BioBERT-v1.1 through a series of extensive experiments on a diverse set of biomedical datasets and tasks. The analyses show that our models are efficient compressed models that can be trained significantly faster and with far fewer parameters compared with their larger counterparts, with minimal performance drops on different biomedical tasks. To the best of our knowledge, this is the first attempt to specifically focus on training compact models on biomedical corpora and by making the models publicly available we provide the community with a resource to implement powerful specialized models in an accessible fashion.

The contributions of this article can be summarized as follows:

- We are the first to specifically focus on training compact biomedical models using distillation and continual learning.
- Utilizing continual learning via the masked language modelling (MLM) objective, we further train three widely used pre-trained compact models, namely DistilBERT, MobileBERT and TinyBERT for 200 K steps on the PubMed dataset.
- We distil three students from a biomedical teacher (BioBERT-v1.1) using three different distillation procedures, which generated the following models: DistilBioBERT, TinyBioBERT and CompactBioBERT.
- We evaluate our models on a wide range of biomedical NLP tasks that include Named Entity Recognition (NER), Question Answering (QA) and Relation Extraction (RE).
- We make all of our six compact models freely available on Huggingface and Github. These models cover a wide range of parameter sizes, from 15 M parameters for the smallest model to 65 M for the largest.

## 2 Background

Pre-training followed by fine-tuning has become a standard procedure in many areas of NLP and forms the backbone for most state-of-the-art models such as BERT (Devlin *et al.*, 2019) and GPT-3 (Brown *et al.*, 2020). The goal of language model pre-training is to acquire effective in-context representations of words based on a large corpus of text, such as Wikipedia. This process is often self-supervised, which means that the representations are learnt without using human-provided labels. There are two main strategies for self-supervised pre-training, namely, MLM and causal language modelling (CLM). In this work, we focus on models pre-trained with the MLM objective.

### 2.1 Masked language modelling

MLM is the process of randomly omitting portions of a given text and having the model predict the omitted portions. The masking percentage is normally 15%, with an 80% probability that the selected word will be substituted with a specific mask token (e.g. <MASK>) and a 20% chance that it will be replaced with another random word (Devlin *et al.*, 2019). Contextualized representations generated using these pre-trained language models are referred to as bidirectional, which means that information from previous and following contexts is used to construct representations for each given word.

### 2.2 BERT: Bidirectional encoder representation from transformers

The most prominent transformer pre-trained with MLM is BERT. BERT is an encoder-only transformer that relies on the multi-head attention mechanism for learning in-context representations. BERT has different variations such as ${\text{BERT}}_{\text{base}}$ and ${\text{BERT}}_{\text{large}}$ which vary in the number of layers and the size of the hidden dimension. Original BERT is trained on English Wikipedia and BooksCorpus datasets for about 1 million training steps, making it a strong model for various downstream NLP tasks.

### 2.3 BioBERT and other biomedical models

While generic pre-trained language models can perform reasonably well on a variety of downstream tasks even in domains other than those on which they have been trained, in recent years researchers have shown that continual learning and pre-training of language models on domain-specific corpora lead to noticeable performance boosts compared with simple fine-tuning. BioBERT is an example of such a domain-specific BERT-based model and the first that is trained on biomedical corpora.

BioBERT takes its initial weights from ${\text{BERT}}_{\text{base}}$ (pre-trained on Wikipedia + Books) and is further pre-trained using the MLM objective on the PubMed and optionally PMC datasets. BioBERT has shown promising performance in many biomedical tasks including NER, RE and QA. Aside from BioBERT, numerous additional models have been trained entirely or partially on biomedical data, including ClinicalBERT (Huang *et al.*, 2019), SciBERT (Beltagy *et al.*, 2019), BioMedRoBERTa (Gururangan *et al.*, 2020) and BioELECTRA (Kanakarajan *et al.*, 2021).

### 2.4 Knowledge distillation

Knowledge distillation (Hinton *et al.*, 2015) is the process of transferring knowledge from a larger model called ‘teacher’ to a smaller one called ‘student’ using the larger model’s outputs as soft labels. Distillation can be done in a task-specific way where the pre-trained model is first fine-tuned on a task and then the student attempts to imitate the teacher network. This is an effective method; however, fine-tuning of a pre-trained model can be computationally expensive. Task-agnostic distillation, on the other hand, allows the student to mimic the teacher by looking at its masked language predictions or intermediate representations. The student can subsequently be directly fine-tuned on the final task (Wang *et al.*, 2020; Yao *et al.*, 2021).

DistilBERT is a prominent example of a compressed model that uses knowledge distillation to transfer the knowledge within the ${\text{BERT}}_{\text{base}}$ model to a much smaller student network which is about 40% smaller and 60% faster. It uses a triple loss which is a linear combination of language modelling, distillation and cosine-distance losses.

## 3 Approach

**Fig. 1.**
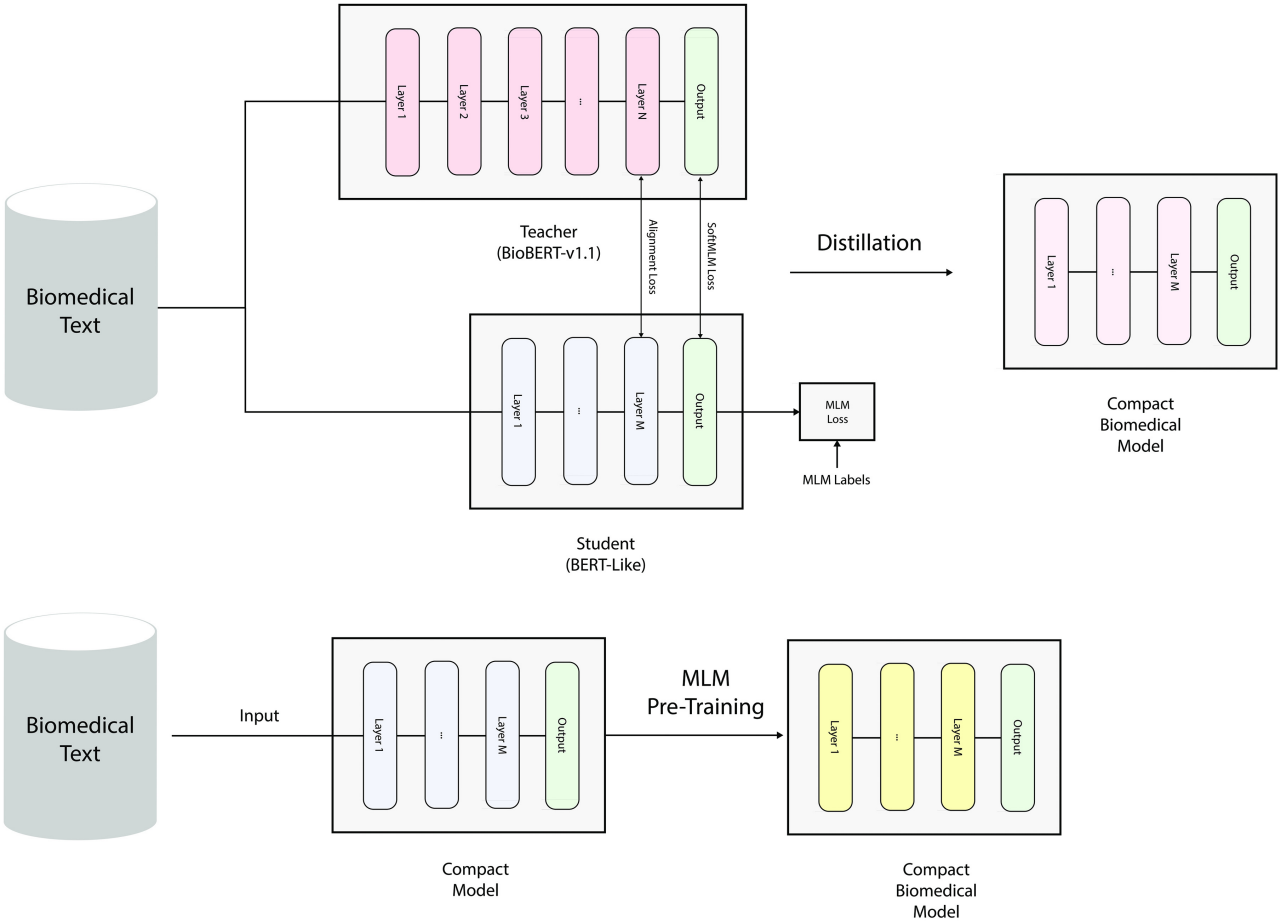
The two general strategies proposed for training compact biomedical models. The first approach is to directly distil a compact model from a biomedical teacher which in our work is BioBERT-v1.1. The distillation depicted in this figure is the same technique used for obtaining DistilBioBERT. TinyBioBERT and CompactBioBERT, on the other hand, employ different approaches, which are not shown here. The second method involves additionally pre-training a compact model on biomedical corpora. For this approach, we use compact models which have been distilled from powerful teachers, namely, DistilBERT (Sanh *et al.*, 2019), TinyBERT (Jiao *et al.*, 2020) and MobileBERT (Sun *et al.*, 2020)

In this work, we focus on training compact transformers on biomedical corpora. Among the available compact models in the literature, we use DistilBERT, MobileBERT and TinyBERT models which have shown promising results in NLP. We train compact models using two different techniques as shown in Figure 1. The first is continual learning of pre-trained compact models on biomedical corpora. In this strategy, each model is further pre-trained on the PubMed dataset for 200 K steps via the MLM objective. The obtained models are named BioDistilBERT, BioMobileBERT and BioTinyBERT.

For the second strategy, we employ three distinct techniques: the DistilBERT and TinyBERT distillation processes, as well as a mixture of the two. The obtained models are named DistilBioBERT, TinyBioBERT and CompactBioBERT. We test our models on three commonly researched biomedical tasks and compare them with BioBERT-v1.1 as shown in Tables 2–7.

## 4 Materials and methods

In this section, we describe the internal architecture of each compact model that is explored in the article, the method used to initialize its weights and the distillation procedure employed to train it.

### 4.1 DistilBioBERT

For distillation, this model employs three losses: MLM, output and alignment. The MLM is a typical MLM loss, as defined below: where *X* is the input, *Y* is the collection of MLM labels, *N* is the number of input tokens and |*V*| is the vocabulary size of the model. Finally, $f_{s}(X)$ represents the student model whose output is *N* probability distribution vectors with size |*V*|. The output loss is defined as a KL divergence between the output distributions of the teacher and student: where $f_{t}(X)$ represents the teacher and $W_{n}$ is a coefficient that ensures that only masked tokens contribute to the computation of loss. The alignment loss is a cosine embedding loss between the last hidden states of the student and the teacher which is mathematically defined as below: where $h_{s}(X)$ and $h_{t}(X)$ output the last hidden states belonging to the student and teacher, respectively, and ϕ(.) is the cosine similarity function. Finally, the overall distillation loss used in this work can be defined as follows: where $\lambda_{1}$ to $\lambda_{3}$ are hyperparameters, controlling the importance of each component of the loss.


(1) $$L_{\text{mlm}}(X,Y)=-\sum_{n=1}^{N}\sum_{i=1}^{\left|V\right|}Y_{i}^{n}\text{ln}(f_{s}{\left(X\right)}_{i}^{n}),$$

(2) $$L_{\text{output}}(X)=\sum_{n=1}^{N}W_{n}D_{\text{KL}}(f_{s}{\left(X\right)}^{n}\;\;||\;\;f_{t}{\left(X\right)}^{n}),$$

(3) $$L_{\text{align}}(X)=\frac{1}{N}\sum_{n=1}^{N}1-\phi (h_{s}{\left(X\right)}^{n},h_{t}{\left(X\right)}^{n}),$$

(4) $$\begin{array}{c} L(X,Y)=\lambda_{1}L_{\text{mlm}}(X,Y)\\ +\lambda_{2}L_{\text{output}}(X)\\ +\lambda_{3}L_{\text{align}}(X) \end{array},$$

#### 4.1.1 Architecture

In this model, the size of the hidden dimension and the embedding layer both set to 768. The vocabulary size is 28 996 for the cased version which is the one employed in our experiments. The number of transformer layers is 6 and the expansion rate of the feed-forward layer is 4. Overall, this model has around 65 million parameters.

#### 4.1.2 Initialization of the student

Effective initialization of the student model is critical due to the size of the model and the computational cost of distillation. As a result, there are numerous techniques available for initializing the student. One method introduced by Turc *et al.* (2019) is to initialize the student via MLM pre-training and then perform distillation. Another approach, which we have followed in this work, is to take a subset of the larger model by using the same embedding weights and initializing the student from the teacher by taking weights from every other layer (Sanh *et al.*, 2019). With this approach, the hidden dimension of the student is restricted to that of the teacher model.

### 4.2 TinyBioBERT

This model uses a unique distillation method called ‘transformer-layer distillation’ which is applied on each layer of the student to align the attention maps and the hidden states of the student with those of the teacher. It employs three losses in total: layer-to-layer alignment loss, output loss and an optional embedding loss. The layer-to-layer alignment loss is used to align the hidden layers of the student and teacher, and it is mathematically defined as follows: where *L* is the number of hidden layers in the student model, $\lambda_{l}$ is a hyperparameter that controls the importance of alignment loss in the *l*th layer, g(.) is a mapping function that maps each student layer to a specific layer of the teacher and MSE(.) is the mean-squared error. $h_{s}^{l}(X)$, $a_{s}^{l}(X)$, $h_{t}^{g(l)}(X)$ and $a_{t}^{g(l)}(X)$ output the hidden states and the attention maps belonging to the lth layer of the student and the g(l)th layer of the teacher. Finally, $W_{p}$ is a projection weight used when the hidden dimensions of the teacher and the student are different. The output loss used in this work is similar to Equation (2). However, instead of KL divergence, the cross entropy loss is employed here, hence this equation is denoted as $L_{\text{output}}^{*}(X)$. The optional embedding loss used when the hidden dimension of the teacher and student differ is defined as follows: where $e_{s}(X)$ and $e_{t}(X)$ output the embedding vectors belonging to student and teacher, respectively. The combined loss used for distilling this model can be formulated as where $\lambda_{0}$ and $\lambda_{L+1}$ are hyperparameters.


(5) $$\begin{array}{c} L_{\text{layer}}(X)=\sum_{l=1}^{L}\lambda_{l}(\text{MSE}(h_{s}^{l}(X)W_{p},h_{t}^{g(l)}(X))\\ +\text{MSE}(a_{s}^{l}(X),a_{t}^{g(l)}(X))) \end{array},$$

(6) $$L_{\text{embed}}(X)=\text{MSE}(e_{s}(X)W_{p},e_{t}(X)),$$

(7) $$\begin{array}{c} L(X)=\lambda_{0}L_{\text{embed}}(X)\\ +L_{\text{layer}}(X)\\ +\lambda_{\left(L+1\right)}L_{\text{output}}^{*}(X) \end{array},$$

#### 4.2.1 Architecture

This model is a 4-layer transformer that uses a hidden dimension and embedding size of 312. The general TinyBERT trained on the Wikipedia uses an uncased tokenizer with a vocabulary size of around 30.5 K words. Hence, for continual learning of the TinyBERT, the uncased tokenizer is used. However, as BioBERT showed the cased tokenizer works better in the biomedical domain, we use a cased tokenizer with a vocabulary size of 28 996 for distilling this model. Overall, both versions have around 15 M parameters.

#### 4.2.2 Initialization of the student

The weight initialization of this model is random since the hidden and the embedding size of this model differ from its teacher. However, the weight initialization of the DistilBERT can be used when the hidden and embedding size of the student are the same as the ones in the teacher which to the best of our knowledge was not tried in the original paper.

### 4.3 CompactBioBERT

This model has the same overall architecture as DistilBioBERT, with the difference that here we combine the distillation approaches of DistilBioBERT and TinyBioBERT. We utilize the same initialization technique as in DistilBioBERT and apply a layer-to-layer distillation with three major components, namely, MLM, compact and output distillation. The compact loss, which distinguishes this model from DistilBERT, is mathematically stated as follows: where *H* is the number of attention heads in the student and teacher. This model’s combined distillation loss is defined as follows: where $\lambda_{1}$ to $\lambda_{3}$ are hyperparameters, controlling the importance of each component of the distillation loss.


(8) $$\begin{array}{c} L_{\text{compact}}(X)=\sum_{l=1}^{L}(\frac{1}{N}\sum_{n=1}^{N}1-\phi (h_{s}^{l}{\left(X\right)}^{n},h_{t}^{g(l)}{\left(X\right)}^{n})\\ +\frac{1}{\text{HN}}\sum_{h=1}^{H}\sum_{n=1}^{N}D_{\text{KL}}(a_{s}^{l}{\left(X\right)}_{n}^{h}\;\;||\;\;a_{t}^{g(l)}{\left(X\right)}_{n}^{h})) \end{array},$$

(9) $$\begin{array}{c} L(X,Y)=\lambda_{1}L_{\text{mlm}}(X,Y)\\ \;\;\;\;\;\;+\lambda_{2}L_{\text{compact}}(X)\\ \;\;\;\;\;\;+\lambda_{3}L_{\text{output}}(X) \end{array}$$

### 4.4 BioMobileBERT

MobileBERT (Sun *et al.*, 2020) is a compact model that uses a unique design comprised of different components to reduce the model’s width (hidden size) while maintaining the same depth as ${\text{BERT}}_{\text{large}}$ (24 transformer layers). MobileBERT has proved to be competitive in many NLP tasks while also being efficient in terms of both computational and parameter complexity. For distillation, MobileBERT uses a layer-to-layer approach which is intended to align the attention maps and hidden states of each student layer with its associated teacher.

#### 4.4.1 Architecture and initialization

MobileBERT uses a 128-dimensional embedding layer followed by 1D convolutions to up-project its output to the desired hidden dimension expected by the transformer blocks. For each of these blocks, MobileBERT uses linear down-projection at the beginning of the transformer block and up-projection at its end, followed by a residual connection originating from the input of the block before down-projection. Because of these linear projections, MobileBERT can reduce the hidden size and hence the computational cost of multi-head attention and feed-forward blocks. This model additionally incorporates up to four feed-forward blocks in order to enhance its representation learning capabilities. Thanks to the strategically placed linear projections, a 24-layer MobileBERT (which is used in this work) has around 25 M parameters. To the best of our knowledge, MobileBERT is initialized from scratch.

## 5 Experiments and results

### 5.1 Task definitions

We test our models in three standard NLP tasks: NER, RE and QA. For each task, a brief description is provided below.

NER is a standard task in NLP and biomedical text mining. In this task, a model is given a sentence and must predict the type of entity that each word in the sentence represents. These entities could denote people, organizations, locations and more. In the biomedical domain, entities may include diseases, genes, species and others.

RE involves predicting the relationship between two entities in a given sentence. In the biomedical domain, examples of RE include identifying the relationship between a gene and a disease or the relationship between a chemical and a protein.

QA is a widely studied task in NLP that involves generating a response to a question posed in natural language. It can be tackled in a generative setting where a question is given to a generative model like GPT-3 (Brown *et al.*, 2020) and it generates an answer based on the data it have been trained on. However, since we do not use generative models in this work, QA here is framed as an extractive task, where a question and a context that contain the answer are provided to the model. The model then learns to predict the span of the context that contains the answer to the question.

### 5.2 Datasets

For biomedical NER, we use eight established datasets, namely, NCBI-disease (Doğan *et al.*, 2014), BC5CDR (disease and chem) (Li *et al.*, 2016), BC4CHEMD (Krallinger *et al.*, 2015), BC2GM (Smith *et al.*, 2008), JNLPBA (Kim *et al.*, 2004), LINNAEUS (Gerner *et al.*, 2010) and Species-800 (Pafilis *et al.*, 2013) which will test the biomedical knowledge of the models in different categories such as disease, drug/chem, gene/protein and species.

For RE, we use the GAD (Bravo *et al.*, 2015) and CHEMPROT (Krallinger *et al.*, 2017) datasets and follow the same pre-processing used in Lee *et al.* (2020). For the GAD dataset, we randomly select 10% of the data for the test set using a constant seed and use the rest for training.

For QA, we train and test on the BioASQ 7b dataset (Tsatsaronis *et al.*, 2015) and follow the same pre-processing steps as Lee *et al.* (2020).

Additional details about these datasets, such as their size and the type of annotations they contain, can be found in Table 1.

**Table 1.** Description of the datasets used in the experiments

Dataset	Task type	Dataset size	Description
NCBI-disease	NER	7287	Dataset collected from 793 PubMed abstracts. It is annotated with disease mentions and concepts.
BC5CDR (disease/chem)	NER	13 938	Corpus constructed from 1500 PubMed articles containing annotations for chemicals and chemical–disease interactions.
BC4CHEMD	NER	87 685	A collection of abstracts from PubMed annotated for chemical entities.
BC2GM	NER	20 131	Dataset consisting of sentences annotated for gene mentions.
JNLPBA	NER	22 402	Dataset collected from MEDLINE abstracts containing annotations for gene entities.
LINNAEUS	NER	23 155	A dataset for species name identification in biomedical domain.
Species-800	NER	8193	A corpus collected from 800 PubMed abstracts and annotated for species entities.
GAD	RE	5330	GAD is a corpus of gene–disease associations.
CHEMPROT	RE	10 065	Dataset from 1820 PubMed abstracts annotated for chemical–protein interactions.
BioASQ 7 b	QA	2747	A QA dataset constructed from a collection of biomedical articles.

### 5.3 Experimental setup

We evaluate our models on three biomedical tasks, namely, NER, QE and RE. For a fair comparison, we fine-tune all of our models using a constant shuffling seed.^1^ Note that the results obtained in this work are for comparison with BioBERT-v1.1 in a similar setting and we are not focusing on reproducing or outperforming state-of-the-art on any of the datasets since that is not the objective of this work.

We distil our students solely from BioBERT and also compare our continually learnt models with it. While there are other recent biomedical transformers available in the literature (Section 1), BioBERT is the most general (trained on large biomedical corpora for 1 M steps) and is widely used as a backbone for building new architectures. Direct comparison with one major model helps us to keep the work focused on compression techniques and assessing their efficiency in preserving information from a well-performing and reliable teacher. These experiments can in the future be expanded to cover other biomedical models.

For NER, all of our models were trained for five epochs with a batch size of 16 and a learning rate of 5e−5. In a few cases, a learning rate of 3e−5 and a batch size of 32 were also used. Because our models contain word-piece tokenizers which may split a single word into several sub-word units, we assigned each word’s label to all of its sub-words and then fine-tuned our models based on the new labels. As shown in Table 2, DistilBioBERT and CompactBioBERT outperformed other distilled models on all the datasets. Among the continually learnt models, BioDistilBERT and BioMobileBERT fared best (Table 3), while TinyBioBERT and BioTinyBERT were the fastest and most efficient models.

**Table 2.** Test results for the models that were directly distilled from the BioBERT-v1.1 on NER datasets

Type	Dataset	Metrics	DistilBERT	DistilBioBERT	CompactBioBERT	TinyBioBERT^a^	BioBERT-v1.1
Disease	NCBI disease	F1	86.38	87.93	**88.67**	85.22	88.62
	BC5CDR	F1	82.01	85.42	85.38	81.28	**86.67**
Drug/chem.	BC5CDR	F1	92.50	94.53	94.31	92.20	**94.73**
	BC4CHEMD	F1	89.53	91.77	91.40	89.03	**92.14**
Gene/protein	BC2GM	F1	84.61	86.60	86.71	82.52	**87.62**
	JNLPBA	F1	79.14	79.97	79.88	78.75	**80.33**
Species	LINNAEUS	F1	80.73	83.29	82.90	78.29	**83.96**
	Species-800	F1	72.03	74.72	75.70	69.59	**77.87**

a Any direct comparison should take into account the fact that other models include over 60 M parameters, whereas TinyBioBERT has only 15 M. Note that the bold numbers denote the best results and the underscored numbers denote the second best results.

**Table 3.** NER test results for models that were pre-trained on the PubMed dataset via the MLM objective and continual learning

Dataset	Metrics	DistilBERT	TinyBERT	MobileBERT	BioDistilBERT	BioTinyBERT	BioMobileBERT
NCBI disease	F1	86.38	80.46	86.14	**87.61**	82.95	87.21
BC5CDR (disease)	F1	82.01	77.45	81.99	**85.61**	81.16	84.62
BC5CDR (chem)	F1	92.50	88.50	92.20	**94.48**	90.85	94.23
BC4CHEMD	F1	89.53	83.76	89.60	**91.59**	87.37	91.31
BC2GM	F1	84.61	76.93	82.86	**86.97**	80.57	85.26
JNLPBA	F1	79.14	76.79	78.88	79.10	77.87	**80.13**
LINNAEUS	F1	80.73	71.94	78.53	**82.56**	76.42	81.83
Species-800	F1	72.03	66.33	74.56	74.68	70.68	75.22

*Note*: The models beginning with the prefix ‘Bio’ are pre-trained, while the rest are baselines.

Bold numbers denote the best performance and underlined numbers denote the second-best performance.

For RE, we trained all of our models for three epochs with learning rates of 5e−5 or 3e−5 and a batch size of 16. CompactBioBERT achieved the best results in both tasks among the distilled models (Table 4), and similarly, BioDistilBERT outperformed all of our continually trained models in both tasks (Table 5).

**Table 4.** Test results of the models that were directly distilled from the BioBERT-v1.1 on RE datasets

Relation	Dataset	Metrics	DistilBERT	DistilBioBERT	CompactBioBERT	TinyBioBERT^a^	BioBERT-v1.1
Gene–disease	GAD	F1	82.54	85.30	85.52	82.46	**86.80**
Protein–chemical	CHEMPROT	F1	47.52	49.79	**52.46**	30.33	52.32

^a^ Any direct comparison between TinyBioBERT and other models should account for the significant difference in model size (15 M versus 60 M). Scores for GAD are in the binary mode and the metrics reported for CHEMPROT are macro-averaged.

Bold numbers denote the best performance and underlined numbers denote the second-best performance.

**Table 5.** Test results on RE datasets for the models that were pre-trained on PubMed via MLM objective and continual learning

Dataset	Metrics	DistilBERT	TinyBERT	MobileBERT	BioDistilBERT	BioTinyBERT	BioMobileBERT
GAD	F1	82.54	75.53	82.98	**86.04**	78.48	84.56
CHEMPROT	F1	47.52	23.18	47.92	**51.48**	25.54	51.03

*Notes*: Model names beginning with the prefix ‘Bio’ are pre-trained and the others are baselines. Scores for GAD are in the binary mode and the metrics reported for CHEMPROT are macro-averaged.

Bold numbers denote the best performance and underlined numbers denote the second-best performance.

For QA, all the models were trained with a batch size of 16. For TinyBERT, TinyBioBERT and BioTinyBERT, a learning rate of 5e−5 was used while for the remaining models this value was set to 3e−5. As seen in Table 6, among our distilled models CompactBioBERT and TinyBioBERT performed best and among our continually learnt models BioMobileBERT and BioDistilBERT outperformed other distilled models (Table 7).

**Table 6.** Test results of the models that were directly distilled from the BioBERT-v1.1 on the BioASQ QA dataset

Dataset	Metrics	DistilBERT	DistilBioBERT	CompactBioBERT	TinyBioBERT^a^	BioBERT-v1.1
BioASQ 7b	S	20.98	20.98	22.83	20.98	**24.07**
	L	29.62	28.39	29.01	30.86	**34.56**
	M	24.34	23.79	25.06	25.05	**28.41**

The metrics used for reporting the results are taken from the BioASQ competition and the models were assessed using the same evaluation script. The metrics are as follows: Strict accuracy (S), lenient accuracy (L) and mean reciprocal rank (M).

Bold numbers denote the best performance and underlined numbers denote the second-best performance.

^a^ Any direct comparison between TinyBioBERT and other models should account for the significant difference in model size (15 M versus 60 M). Scores for GAD are in the binary mode and the metrics reported for CHEMPROT are macro-averaged.

**Table 7.** BioASQ QA test results for the models that were pre-trained on the PubMed dataset via MLM objective and continual learning

Task	Metrics	DistilBERT	TinyBERT	MobileBERT	BioDistilBERT	BioTinyBERT	BioMobileBERT
BioASQ 7b	S	20.98	21.60	27.77	25.92	20.37	**29.01**
	L	29.62	29.62	**40.74**	38.88	32.09	38.88
	M	24.34	24.62	32.78	30.83	25.20	**32.90**

*Notes*: The metrics used for reporting the results are taken from the BioASQ competition and the models were assessed using the same evaluation script. The metrics are as follows: Strict accuracy (S), lenient accuracy (L) and mean reciprocal rank (M) scores.

Bold numbers denote the best performance and underlined numbers denote the second-best performance.

## 6 Discussion

In this study, we investigated two approaches for compressing biological language models. The first strategy was to distil a model from a biomedical teacher and the second was to use MLM pre-training to adapt an already distilled model to a biomedical domain. Due to computational and time constraints, we trained our distilled models for 100 K steps and our continually learnt models for 200 K steps; as a result, directly comparing these two types of models may be unfair. We observed that distilling a compact model from a biomedical teacher increases its capacity to perform better on complex biomedical tasks while decreasing its general language understanding and reasoning. This means that while our distilled models perform exceptionally well on biomedical NER and RE (Tables 2 and 4), they perform comparatively poorly on tasks that require more general knowledge and language understanding such as biomedical QA (Table 6).

Weaker results on QA (compared with continually learnt models) suggest that by distilling a model from scratch using a biomedical teacher, the model may lose some of its ability to capture complex grammatical and semantic features while becoming more powerful in identifying and understanding biomedical correlations in a given context (as seen in Table 4). On the other hand, adapting already compact models to the biomedical domain via continual learning seems to preserve general knowledge regarding natural language structure and semantics in the model (Table 7). It should be noted that the distilled models are only trained for 100 K steps and this analysis is based on the current results obtained by these models.

Furthermore, despite having nearly half as many parameters, BioMobileBERT outscored BioDistilBERT on QA. As previously stated, MobileBERT employs a unique structure that allows it to get as deep as 24 layers while maintaining less than 30 M parameters. On the other hand, BioDistilBERT is only six layers deep. Because of this architectural difference, we hypothesize that the increased number of layers in BioMobileBERT allows it to capture more complex grammatical and semantic features, resulting in superior performance in biomedical QA, which requires not only biomedical knowledge but also some general understanding about natural language.

We trained models of varied sizes and topologies, ranging from small models with only 25 M parameters to larger models with up to 65 M. In our experiments, we discovered that when fine-tuned with a high learning rate (e.g. 5e−5), our tiny models, TinyBioBERT and BioTinyBERT, perform well on downstream tasks while our bigger models tend to perform better with a lower learning rate (e.g. 3e−5).

In addition, we found that compact models that have been trained on the PubMed dataset for fewer training steps (e.g. 50 K) tend to achieve better results on more general biomedical datasets such as NCBI disease which are annotated for disease mentions and concepts and perform worse on more specialized datasets like BC5CDR-disease and BC5CDR-chem which include extra domain-specific information (e.g. chemicals and chemical–disease interactions), and the reverse is true for the models that are trained longer on the PubMed dataset.

**Fig. 2.**
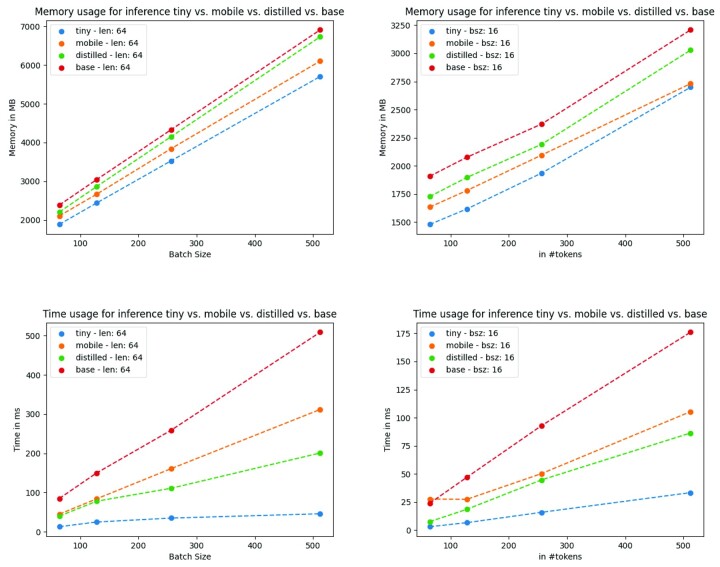
The inference time/memory comparison of our proposed models. ‘Small’ refers to TinyBioBERT, ‘mobile’ to BioMobileBERT, ‘distilled’ to DistilBioBERT and CompactBioBERT (since they share the same architecture) and ‘base’ to BioBERT-v1.1

TinyBioBERT and BioTinyBERT are the most efficient models in terms of both memory and time complexity (as evidenced in Fig. 2). DistilBioBERT, CompactBioBERT and BioDistilBERT are the second most efficient set of models in terms of time complexity. BioMobileBERT, on the other hand, is the second most efficient model with regards to memory complexity. In conclusion, if efficiency is the most important factor, the tiny models are the most suitable resources to use. In other use cases, we recommend either the distilled models or BioMobileBERT depending on the relative importance of memory, time and accuracy.

## 7 Conclusion

Lightweight models developed here can either be used in isolation for tasks and scenarios where either computational resources are limited or when a small drop in performance would be an acceptable trade-off for faster processing. Another scenario is when a compact model can be used as a lightweight front-end for a larger model, with the larger model only being used to handle cases where the compact model is not confident or where more detailed analysis is needed. This approach allows the larger model to be used when necessary, while also leveraging the benefits of a fast and compact model.

In this work, we employed a number of compression strategies to develop compact biomedical transformer-based models that proved competitive on a range of biomedical datasets. We introduced six different models ranging from 15 M to 65 M parameters and evaluated them on three different tasks. We found that competitive performance may be achieved by either pre-training existing compact models on biomedical data or distilling students from a biomedical teacher. The choice of distillation or pre-training is dependent on the task, since our pre-trained students outperformed their distilled counterparts in some tasks and vice versa.

We discovered, however, that distillation from a biomedical teacher is generally more efficient than pre-training when using the same number of training steps. Due to computational and time constraints, we trained all of our distilled models for 100 K steps, and for continual learning, we trained models for 200 K steps. For future work, we plan to pre-train models for 500 K to 1 M steps and publicly release the new models. In addition, since CompactBioBERT and DistilBioBERT performed similarly on most of the tasks, we plan to investigate the effect of hyperparameters on training these models in order to determine which distillation technique is more efficient. Some of the compact biomedical models proposed in this study may be used for inference on mobile devices, which we hope will open new avenues for researchers with limited computational resources.

## Data Availability

The datasets were derived from sources in the public domain. All of the NERs + GAD: http://nlp.dmis.korea.edu/projects/biobert-2020-checkpoints/datasets.tar.gz. ChemProt: https://huggingface.co/datasets/zapsdcn/chemprot. BioAsq 7b: https://drive.google.com/file/d/1-KefyBWOaCuswy9LFwnq7NC0H1Ymkv05/view.
